# Electrochemical (Bio)Sensors Based on Covalent Organic Frameworks (COFs)

**DOI:** 10.3390/s22134758

**Published:** 2022-06-23

**Authors:** Emiliano Martínez-Periñán, Marcos Martínez-Fernández, José L. Segura, Encarnación Lorenzo

**Affiliations:** 1 Departamento de Química Analítica y Análisis Instrumental, Facultad de Ciencias, Universidad Autónoma de Madrid, 28049 Madrid, Spain; emiliano.martinez@uam.es; 2 Departamento de Química Orgánica I, Facultad de CC. Químicas, Universidad Complutense de Madrid, 28040 Madrid, Spain; marcma09@ucm.es; 3 Institute for Advanced Research in Chemical Sciences (IAdChem), Facultad de Ciencias, Universidad Autónoma de Madrid, 28049 Madrid, Spain; 4 IMDEA-Nanociencia, Ciudad Universitaria de Cantoblanco, 28049 Madrid, Spain

**Keywords:** COF, electrochemical biosensors, electrochemical sensors

## Abstract

Covalent organic frameworks (COFs) are defined as crystalline organic polymers with programmable topological architectures using properly predesigned building blocks precursors. Since the development of the first COF in 2005, many works are emerging using this kind of material for different applications, such as the development of electrochemical sensors and biosensors. COF shows superb characteristics, such as tuneable pore size and structure, permanent porosity, high surface area, thermal stability, and low density. Apart from these special properties, COF’s electrochemical behaviour can be modulated using electroactive building blocks. Furthermore, the great variety of functional groups that can be inserted in their structures makes them interesting materials to be conjugated with biological recognition elements, such as antibodies, enzymes, DNA probe, aptamer, etc. Moreover, the possibility of linking them with other special nanomaterials opens a wide range of possibilities to develop new electrochemical sensors and biosensors.

## 1. Introduction

**Figure 1:**
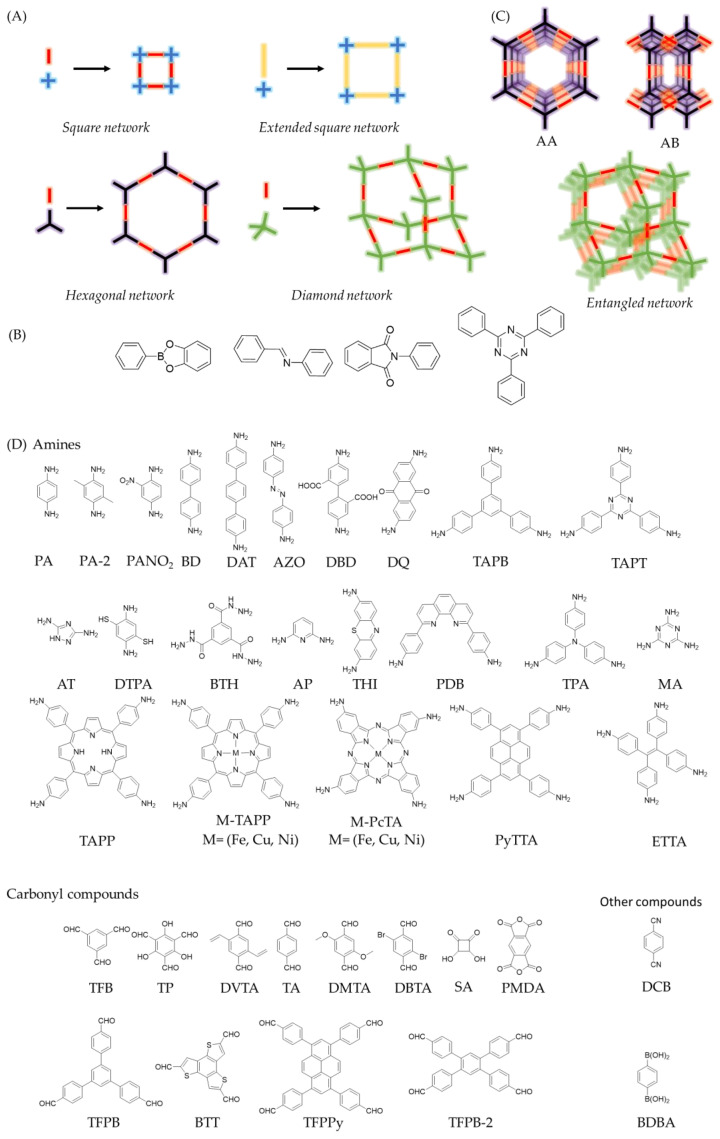
(**A**) Examples of COFs topology diagrams. (**B**) Examples of linkages commonly used for the synthesis of COFs (from left to right: boronate ester, imine, imide, and triazine. (**C**) Examples of arrangements of COF’s networks. (**D**) List of linkers named in this review.

Covalent organic frameworks (COFs) are a type of polymer, which connect organic molecules in two (2D) or three dimensions (3D) via covalent bonds [1,2]. Their most noteworthy feature is their intrinsic order, which is predetermined by the monomers or linkers, employed in the polymerization, building crystalline structures with pre-designable architectures [3,4,5]. The design of the network’s topology lies in the control of the direction of covalent bond formation during the polymerization [1]. To ensure the directionality of growth, the monomers must be formed by relatively rigid bonds and present the functionalities in specific positions [6,7]. To do this, the design is approached from a simplified block model, where each monomer is represented with a specific geometric shape that symbolizes the relative positions of the reactive points [6,7,8]. In this way, as depicted in Figure 1A, a linear monomer could generate hexagonal networks by cyclotrimerization (for example boroxine formation) or produce square COFs if it is combined with a C_4_ linker. Following the pioneering work of Yaghi and the co-workers of Yaghi [9], several structural motifs have been described, including hexagonal [10,11], kagome [12,13,14] or square [15,16] two-dimensional networks (2D-COFs), and diamonoid [17,18], Cubic [19], or *PtS* [20] three-dimensional architectures (3D-COFs). It is worth pointing out that the dimensionality of the COF network is also determined by the linkers employed in the polymerization. The aforementioned 2D-COFs are generally built from flat molecules, while 3D-COFs are usually based on linkers endowed with sp^3^ centres [18] or a geometry significantly distorted from planarity via steric tuning [20].

The type of bond used to connect the building blocks, commonly known as linkage, is crucial during the formation of the framework as multiple covalent structures with different degrees of crystallinity can be produced during the polymerization reactions [6,7]. In fact, the main difference between COFs and porous organic polymers (POPs) is the crystalline order. The generally most widespread strategy to prevent the formation of amorphous products and favour the COF’s crystallization is the use of reversible reactions [6,7]. In this way, the balance between reactants and products allows curing and error correction of the amorphous network, usually associated to kinetic control products, until the COF crystallizes as the thermodynamically more stable product [7]. These are the characteristic features of dynamic covalent chemistry (DCC) [21,22]. In simple terms, in a system where DCC operates, it is the stability of the products that decides the final distributions [2]. Thus, several linkages have been used for the formation of COFs, such as boroxine, boronate esterification, imine, azine, imide, or triazine (Figure 1B) [6]. Other approaches to enhance the crystallinity of the systems includes the addition of monofunctional modulators [18], competitors [23], and protected monomers [24] as strategies to control the nucleation and growth of the crystalline phases [25,26]. Unfortunately, the crystallization conditions to obtain the more thermodynamically stable products are not known *a priori*, and the COF crystallization is usually achieved via iterative screening methodology. Nevertheless, a plethora of methodologies have been reported in the last years to enhance the crystallinity of COFs, including the solvothermal synthesis [10], microwave-assisted methods [27], ionothermal synthesis [28], interfacial synthesis [29], repolymerization methods [30], or on-surface synthesis [31]. Furthermore, the screening of experimental conditions and structure–property relationships are being accelerated through computational calculations and automated machine learning [32,33,34], which are emerging as powerful tools to obtain tailor-made materials in record times.

Another distinctive characteristic of COFs is their inherent porosity [35,36,37]. On the one hand, the overlap of the 2D-COFs layers can build up a three-dimensional structure, traversed by unidirectional cavities also known as pores, which can mainly present as two isomers. The first one is an eclipsed structure (AA), composed by adjacent layers, which are located in the same position. The second one, is a staggered structure (AB or ABC), where every COF layer is shifted with respect to the previous one. It is worth pointing out that the staggered structure in 2D-COFs also presents pores traversing the three-dimensional structure. However, in terms of porosity, the structure that presents the highest surface area is the eclipsed isomer network [1,2]. On the other hand, network entanglements can be produced in 3D-COFs, thus lowering the theoretical surface area (Figure 1C) [18].

COFs presents high thermal, mechanical, and chemical stabilities, with differences depending on the linkage and/or the monomers used in the polymerization reaction [6]. The above-mentioned characteristics, together with their inherent insolubility [1], place COFs as ideal materials to explore their applicability in heterogeneous phases. In this way, COFs have already been used in different applications, such as catalysis [38,39,40], solar cells [41], batteries [42,43], gas storage and separation [44,45], water remediation [39,46], and sensing [47].

Concerning the integration in devices, COFs must face the doble edged sword of one of their main attributes, their inherent insolubility. In this way, the most common procedure to achieve tailor-made devices are two depending on the followed path: (i) Top-down protocol involves the destruction of the granular COF structure, achieving the sheet separation in a process known as exfoliation producing covalent organic nanosheets (CONs) [48]. To address this end, several methods have been developed. The most common procedure is liquid phase exfoliation, which consists in the use of different solvents to produce layer slipping and the subsequent delamination in an ultrasonic bath [49]. Another method is the mechanical delamination, which produces the exfoliation by using an external force over the bulk COF [50]. Chemical or acid spontaneous exfoliations are receiving a great deal of attention because they cause the COF delamination using chemical energy, reducing the costs for obtaining CONs [49]. Finally, nitrogen delamination consists in the gentle heating of the bulk COF and subsequent addition of liquid nitrogen, which intercalates between the COF layers and produces exfoliation by the thermal expansion in the liquid-to-gas phase transition [51]. (ii) The use of bottom-up methods involves the formation of the CONs or the tailor-made COF directly from the monomers. On the one hand, for the synthesis of nanosheets it usually requires the use of additives, such as tetra-n-butylammonium fluoride (TBAF) micelles or surfactants mediators producing monomodal distribution of CONs by the restriction of the growth dimensions. Thus, mi-cellar synthesis allows the growing of large few-layered COFs in the interphase between two immiscible solvents [29,52]. On the other hand, tailor-shaped COFs are obtained by using either the 3D-printing technique, which involves the preparation of a COF “ink” and the subsequent printing following a layer-by-layer protocol [53,54] or the formation of monolithic COF aerogels by crystallization in moulds to produce tailor-made macroscopic objects [55,56].

Concerning sensing applications, between all the COF-based sensor assemblies, electrochemical sensors present important advantages, such as their high porosity, which results in increased sensibility, and the high specificity and fantastic biocompatibility that improves the stability of the electrochemical sensors [57]. The most common procedure followed to achieve these devices is the use of COFs as supports to anchor the active species (or guests) towards the recognition [58,59]. In this way, the COF enhances the electrochemically active surface area of the electrode by preventing the agglomeration of the active species [59,60]. There are three main methods to anchor the active species into COFs. The first one involves the coordination of the metallic species in Lewis acid or basic positions of the COF [61]. The second one involves the active specie anchorage by other non-covalent interactions, such as π-interactions [59]. Finally, covalent immobilization can also be achieved by chemical reactions involving backbone modification or pendant groups reactions, highlighting *Hüisgen’s* azide–alkyne cycloaddition as the most prominent example of these guests’ immobilization methods [5].

To achieve electrochemical sensing, great conductivity is desired. However, despite COFs presenting an ordered and columnar π-skeleton, the electrochemical sensing is still limited due to the polygranular COF’s morphology, leading to insignificant hopping-type electrical conductivity in most of the examples [62]. In the past few years, several strategies have been developed to address this problem. To avoid this problem, conductive additives can be added during the sensor assembly. The most common example is the in situ reduction of metallic precursors to produce metallic nanoparticles (MNPs), which can be anchored to the COF pore walls by coordinative interactions preventing the NPs agglomeration [63,64]. Other common conductive additives are carbonaceous compounds, such as carbon black (CB) [65], pyrolyzed three-dimensional kenaf stem (3D-KSC) [66,67,68], or multi-wall carbon nanotubes (MWCNTs) [69].

Sensor assemblies are usually achieved by different strategies. One of them involves the mixing of the COF with the conductive additives in a hollow glass tube [70,71]. A second strategy involves the electrode modification with Covalent Organic Nanosheets (also known as CONs [72,73]), produced by delamination of COFs usually by liquid phase exfoliation (LPE) and subsequent drop casting of CONs colloids [74,75,76]. Finally, the sensor’s design can be based on simple electrodes [58,61] of dual systems, highlighting the sandwich-type electrodes [77,78].

Different examples of electrochemical sensors and biosensors are exposed in this review, showing the great versatility of functions and capabilities of materials based on COFs, resulting in interesting approaches that can enhance the sensibility and selectivity of the final devices.

## 2. Electrochemical Sensors

COFs have been widely used in the development of the electrochemical sensor. We have classified COFs in three different groups attending to their main role in electrochemical sensors.

### 2.1. COFs Acting as Electrocatalysts

The easiest configuration for the use of COFs in the development of electrochemical sensors involves the direct deposition of the COF as the modifier on top of a working electrode surface. The high porosity of the COFs-based materials and their crystalline structure make them interesting materials for that purpose, as shown in the work developed by Y.-H. Pang et al. [79]. The COF synthesis is based on the functionalization of 1,3,5-triformylphloroglucinol (**Tp**) with chiral (+)-diacetyl-L-tartaric anhydride to produce the reactive monomer **CTp**. The subsequent condensation of **CTp** with the other reactive monomer, 2,5-dimethyl-*p*-phenylenediamine (**Pa-2**) generates the chiral COF **CTpPa-2**. The COF is suspended in an ethanol/Nafion solution, and the suspension is drop-casted on top of a GC electrode. In this example, the COF improves the electrocatalytic performance through the electrooxidation of bisphenol A and bisphenol S. A similar example is presented by the same research group [80], but in this case using a pencil graphite as working electrode, which is modified by drop casting with β-ketoenamine-linked **COF DQTP**. This COF is synthesized by 1,3,5-triformylphloroglucinol (**TP**) and 2,6-diaminoanthraquinone (**DQ**) by using the solvothermal method. The developed electrochemical sensor exhibited high electrical conductivity and catalytic activity. It has been applied for simultaneous determination of bisphenol A and bisphenol S in food packages with a good linearity range (from 0.5 to 30 μM) for two bisphenols and a detection limit of 0.15 μM (S/N = 3). Another example for the use of a COF material directly deposited on top of the working electrode is reported by P. Arul, et al. [81]. In this case, the COF used is the 3,5-diamino-1,2,4-triazole-COF (**DAT-COF**). The **DAT-COF** is dissolved in 0.1 M H_2_SO_4_ and a film of **DAT-COF** on GC electrode is generated by potential cycling between −0.40 to +1.70 V at a scan rate of 50 mV s^−1^ for 20 cycles. **DAT-COF** film/GCE not only determines simultaneously dihydroxybenzene isomers with detection limits lower than 0.12, (S/N = 3), but also selectively determines one isomer in the presence of the other two isomers.

#### 2.1.1. COFs Combined with Carbon Materials

The main drawback of using COFs as electrode modifiers is their usual insulating character. To avoid this inconvenience, they are usually employed together with carbon conducting materials. In this sense, the easiest way of improving the electron/hole transport is mixing COFs with conductive carbon and fabricating traditional paste carbon electrodes. This strategy has been followed by Hou et al. [71]. **TpBD-COF** was synthesized using 1,3,5-triformylphloroglucinol and benzidine as the precursors. The main advantages of **TpBD-COF** are its high chemical stability, the abundance of functional groups, and its large surface area. This sensor presents the rapid quantitative determination of hydroquinone and catechol by differential pulse voltammetry (DPV). Another approach of the use of COF with conductive carbon materials is described by Zhang et al. [65]. Thus, a triphenylamine-based covalent-organic framework (**TPA-COF**) is composited with carbon black (CB) using a one-pot solvothermal method. A GC electrode can be modified by drop casting the CB-doped **TPA-COF**, suspended in a solution of water: ethanol (50:50) and Nafion. The modified electrode has been successfully applied to the detection of dopamine in real samples.

COFs have also been used in combination with carbon nanomaterials to avoid the lack of electron conductivity. One example is described by Yang et al. [82]. The COF used in this case (**TAPT-TFP-COF**) is prepared by Schiff base condensation reaction between 1,3,5-tris-(4-aminophenyl)triazine (**TAPT**) and 1,3,5-triformyl phloroglucinol (**TFP**). The composite material based on **TAPT-TFP-COF** and carboxyl-functionalized multi-wall carbon nanotubes (**COOH-MWCNTs**) are drop casted on the GC electrode. The modified electrode can be used as an electrochemical sensor for the simultaneous determination of paracetamol and dopamine with detection limits (LOD) of 0.14 μM and 0.19 μM, respectively. **COOH-MWCNTs** interconnect **TAPT-TFP-COF** domains and act as bridges between the COF particles, showing a good synergistic effect and accelerating electron transfer. Another similar approach is described by Y. Sun, et al. [83]. The COF used in this example is synthesized using 1,3,5-tris-(4-aminophenyl)benzene (**TAPB**) and terephthaldicarboxaldehyde (**TPA**) in the presence of **NH_2_-CNT**. The obtained **COF@NH_2_-CNT** possesses good conductivity and high specific surface area, when it is deposited on a GC electrode. The **COF@NH_2_-CNT**/GCE sensor showed great analytical performance for the determination of furazolidone (nitrofuran antibacterial agent). It offered a wide range furazolidone quantification range from 0.2 μM to 100 μM, with a low detection limit of 7.75 × 10^−8^ M.

#### 2.1.2. COFs Combined with Conducting Polymers

Some innovative approaches to improve the conductivity of COFs involves the development of composite materials that combine COFs with conducting polymers [84]. A core–shell material made of **TAPB-DMTP-COF** (**TAPB**, 1,3,5-tris(4-aminophenyl)benzene; **DMTP**, 2,5-dimethoxyterephaldehyde) core and a polyanyline-conducting polymer as shell, **TAPB-DMTP-COF@PANI**, is solvothermally synthesized. A GC electrode is modified by drop casting from a water suspension of the **TAPB-DMTP-COF@PANI**. The modified electrode has been successfully applied for the detection of acetaminophen in commercial tablets, human blood, and urine. The composite material facilitated the interaction of acetaminophen with absorption positions by π–π stacking and hydrogen bonding at the time that improves the COF conductivity. Under the optimal conditions, a detection limit of 0.032 μmol/L and a wide linear range of 0.10–500 μmol/L acetaminophen were obtained. The electrochemical platform was almost unaffected by other interfering substances.

#### 2.1.3. COFs Combined with Metal Oxide Particles

**Figure 2:**
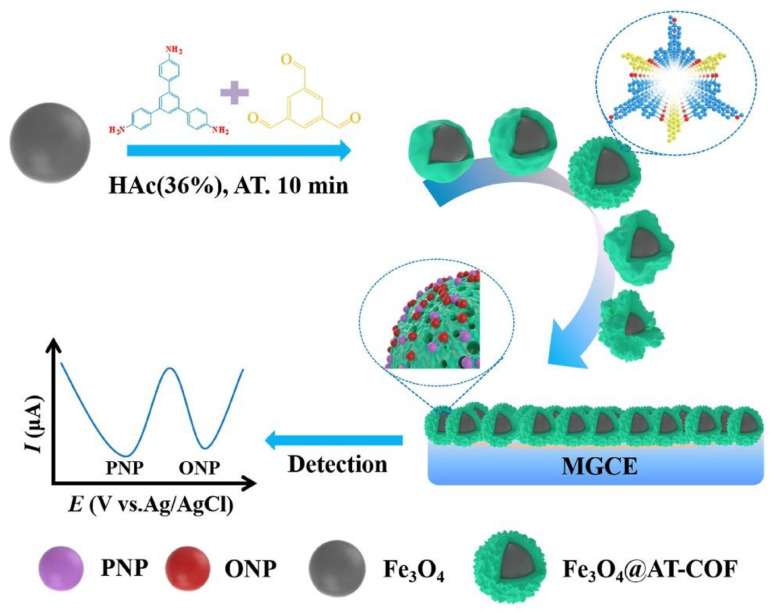
Schematic illustration of a glassy carbon electrode modified with a magnetic **Fe_3_O_4_@AT-COF** nanocomposite used for the simultaneous detection of **PNP** and **ONP**. Reprinted with permission from Ref. [85]. Copyright 2019 Elsevier.

An excellent alternative is the modification of magnetic nanomaterials with COFs instead of directly modifying the working electrode surface. This alternative has been described by Q. Wang, et al. [85] (see Figure 2). The **Fe_3_O_4_@AT-COF** is prepared via a one-pot ambient temperature solution phase method by adjusting the amount of reaction solvent. A mixture of 1,3,5-tris(4-aminophenyl) benzene (**TAPB**) and 1,3,5-benzenetricarboxaldehyde (**TFB**) with Fe_3_O_4_ particles is employed during the synthesis. The obtained **Fe_3_O_4_@AT-COF** exhibits high surface area, good water dispersion, long-term stability, excellent electrical conductivity, and pre-concentration effect. Once the modified magnetic beads are retained in the working electrode surface by a magnet, the electrochemical platform has been used for the simultaneous detection of *p*-nitrophenol (**PNP**) and o-nitrophenol (**ONP**) with a wide linear range from 10 to 3000 μM and the low detection limits of 0.2361 μM and 0.6568 μM, respectively. It can be applied in lake and tap water for monitoring PNP and ONP with outstanding sensitivity and reliability.

#### 2.1.4. CODs with Electroactive Moieties

The great versatility in the syntheses of COFs allows the introduction of electroactive moieties in their structures. Some examples of COFs endowed with electroactive units have been reported, which originates the appearance of redox processes confined on the electrode surface as a consequence of reductions/oxidations within the COF structure itself. The first example reporting an electrochemical sensor using an electroactive COF was described by Y. Song et al. [69]. The COF used was obtained by dehydration condensation reaction between 1,3,5-tris(*p*-formylphenyl)benzene (**TFPB**) and thionine (**Thi**) mixed with carbon nanotubes (CNTs) to prepare **COF-Thi−TFPB−CNT** composites. The GC electrode modification is carried out by drop casting a water suspension prepared with **COF-Thi−TFPB−CNT**. The modified electrode was efficiently used for the determination of ascorbic acid (**AA**) and pH sensing. The three-dimensional porous carbon from kenaf stem (**3D-KSC**) has been also combined with **COF-Thi-TFPB** [66] to prepare a composite material to be used as a carbon paste electrode. The composite **3D-KSC/COF-Thi-TFPB** electrocatalyzes the reduction of riboflavin. Double signals allow measurement correction with the aim to make them more accurate and reliable. As a consequence, the selectivity and reproducibility of the sensor is greatly improved. Another example of an electroactive COF is described by P. Zhu et al. [86]. These authors managed to synthetize a reduced-GO-COF-based composite by in situ polymerization. First, 3D graphene aerogel was prepared by hydrazine reduction of freeze-dried GO. Afterwards, the solvothermal synthesis of the COF was carried out by reaction between 5,10,15,20-tetrakis[(4-aminophenyl) porphinato]-iron [**Fe** (**TAPP**)] and terephthalaldehyde, using 1,3,5-trimethylbenzene, ethanol and aqueous acetic acid as solvents in the presence of the prepared graphene aerogel (**GA**) generating the three-dimensional **COF-366-Fe/GA** composite. The composite was suspended in a mixture of ethanol, water, and Nafion and drop casted on top of the GC electrode. The **COF-366-Fe/GA**-based sensing platform benefits from the remarkable synergy effect between COF-366-Fe and 3D GA, presenting ultrasensitive response to NO in the wide range from 0.18 to 400 μM with a lower detection limit (30 nM) and higher sensitivity (8.8 μA·μM^−1^·cm^−2^). It has been applied for the real-time identification of NO secreted from complex biological systems.

On the other hand, Yonghai Song and col. [87] managed to synthetize a porphyrin-based COF by the Schiff base condensation reaction between 1,3,5-benzenetricarboxaldehyde and 5,10,15,20-tetrakis(4-aminophenyl)-21H,23H-porphyrin to yield **COFp-por NH_2_-BTA**. Subsequent Iron coordination afforded the metalated **COFp-Fepor NH_2_-BTA**. Finally, drop casting was used to produce **COF-p-FePor NH_2_-BTA**/GC electrodes. The modified electrodes were successfully used as H_2_O_2_ and pH electrochemical sensor.

### 2.2. COFs Acting as Support of Electrocatalyst and/or Recognition Elements

One of the main functions of COFs in this context is their role as a recognition element in the sensor. The high porosity of COFs together with the possibility of the delamination of 2D-COFs allow the production of 2D-nanomaterials with enhanced surface–volume ratio, which allows an increment of the sensor recognition element or the electroactive centre exposition to the measuring solution media.

#### 2.2.1. COFs with Metal Nanoparticles and/or Carbon Materials

Simple examples of the joint use of COFs and metal nanoparticles have been also reported. Among them, it is worth highlighting the new squarine-linked COF (TS-COF) obtained by reaction between tris-(4-aminophenyl)-s-triazine (TAPT) and squaric acid (SA) under solvothermal conditions [88]. In order to increase the electrochemical performance, gold nanoparticles (**AuNPs**) were loaded by the in situ reduction of chloroauric acid and reduced graphene oxide (**RGO**) to enhance the electrical conductivity. The **AuNPs@TS-COF/RGO** nanocomposite thus obtained was used as a sensor with an enhanced sensibility due to the synergic properties of **AuNPs**, **TS-COF**, and **RGO**. It has been successfully used for the simultaneous recognition of uric acid, dopamine, and ascorbic acid.

A similar strategy was followed by Guan et al. [64]. They designed a pyrene-based **TF-Py-COF**, which could be obtained by the solvothermal reaction between 1,3,6,8-tetrakis(4-formyl phenyl)-pyrene (**TFPPy**) and 1,3,6,8-tetrakis(4-aminophenyl)-pyrene (**PyTTA**). The electrochemical sensing platform was developed by the physical mixture of **TF-Py-COF** and a 3D carbon material containing nitrogen and **AuNPs**. It was used for the simultaneous detection of acetaminophen and 4-aminophenol. Good electrochemical performance was a consequence of the synergetic effect of the porous structure of the COF, the great conductivity of 3D carbon material containing nitrogen, and the excellent electrocatalytic activity of **AuNPs**.

Other carbon-based materials with high conductivity, such as multiwall carbon nanotubes (**MWCNTs**), have also been used to improve the low conductivity of COFs together with AuNPs, to enhance the electrocatalytic activity of the electrochemical platform [89]. The research group of Wu Yang synthetized a pyrene-based COF by the Schiff base condensation reaction between 1,3,6,8-Tetra (4-formyl phenyl) pyrene (**TFPPY**) and 1,4-phenylenediamine under solvothermal reaction conditions to afford **IL COF-1**. In a second-step, electrode modification was achieved by a first liquid phase exfoliation of the **IL COF-1** in the presence of **MWCNT-NH_2_** and **AuNPs** synthetized in situ, followed by the subsequent drop casting over a GC electrode of the colloid to prepare the **COF-NH_2_-MWCNT**/Au/GC. The porous **IL COF-1** with pyrene groups effectively increases the number of binding sites available on the surface of the electrode. The resulting modified electrode provided excellent electrochemical activity for the simultaneous detection of dopamine and uric acid. Another example of the combination of COF and AuNPs is described by Yang et al. [90]. In this work, the condensation between 1,3,6,8-tetra(4-formyl phenyl) pyrene (**TFPPy**) and 2,6-diaminopyridine yield the new **DP-Py COF** with an unusual wavy topology. This COF presents multiple chelation points for metal nanoparticles, such as the imine linkages or the pyridine moieties. Thus, the prepared **DP-Py COF** was modified with **AuNPs** and drop casted in a GCE to fabricate a novel electrochemical sensor **DP-Py-COF**/**AuNPs**/GCE for sensitive and rapid determination of theophylline and caffeine.

Other research groups use the strong electrostatic interaction of **AuNPs** and the unsaturated amine group present on **TAPB-DMTP-COFs** (**TAPB**, 1,3,5-tris(4-aminophenyl)benzene; **DMTP**, 2,5-dimethoxyterephaldehyde) to successfully immobilize AuNPs in the COF-porous nanostructure [91]. GC electrodes modified by a drop casting deposition of the nanocomposite material show high electrocatalytic activity toward the oxidation of chlorogenic acid. These electrochemical sensors show a wide linear range of 1.0 × 10^−8^–4.0 × 10^−5^ M and a low detection limit of 9.5 × 10^−9^ M, as well as a good repeatability of 4.1% in 2.0 × 10^−5^ M chlorogenic acid.

There also some examples of electrochemical sensors that use silver nanoparticles (**AgNPs**) together with COFs as support materials. Thus, Shankar and co-workers [92] reported the synthesis of a COF by reaction between *p*-phenylenediamine and terephthalaldehyde under reflux (DMF; 150 °C; 12 h). **AgNPs** can be subsequently embedded in the COF from aqueous solutions of AgNO_3_ and NaBH_4_. Finally, GC electrodes drop casted with the obtained suspension were used to electrocatalyze DNA bases oxidation at different potentials, allowing the simultaneous determination of adenine, thymine, guanine, and cytosine by differential pulse voltammetry (DPV).

Platinum nanoparticles (**PtNPs**) have also been employed in electrode configurations similar to that described above [63]. In a representative contribution, Yang and co-workers reported the Schiff base condensation reaction between 1,3,5-tris-(4-formylphenyl) benzene (**TFPB**) and benzidine (**BD**) to yield the TFPB-**BD-COF**. For the sensor assembly, the authors reacted to the aldehyde terminal groups with **NH_2_—MWCNTs** and assembled **PtNPs** by in situ reduction. In this way, the **TFPB-BD-COF**/**PtNPs**/**NH_2_-MWCNTs** offers a large surface area platform thanks to the presence of **TFPB-BD-COF**, with highly dispersed **PtNPs**, which enhance electrocatalytic activity, and high conductivity contributed by the **MWCNTs**. The novel electrochemical sensor is successfully used for the simultaneous detection of catechol, hydroquinone, and resorcinol. Li Wang’s research team described another example of the use of **PtNPs** [67]. Three-dimensional porous carbon (**3D-KSC**) is used during the synthesis of **TAPB-PDA-COF** generating a hybrid material **3D-KSC/COF-TAPB-PDA**. The obtained material is used to prepare a carbon paste electrode, which displays a large specific active surface area. In a second step, NPs loading was achieved by in situ electro-deposition of CuCl_2_ and H_2_PtCl_6_ to produce CuNPs and PtNPs. The **3D-KSC/COFTAPB-PDA/CuNPs** and **3D-KSC/COFTAPB-PDA/PtNPs** showed good analytical parameters for glucose and H_2_O_2_ determination, respectively.

#### 2.2.2. COFs with Metal Oxide Nanomaterials

Metal oxide nanomaterials have also been combined with COFs to provide materials for the development of electrochemical sensors as recently reported by Yang and co-workers [93]. In this work, the reaction between 2,5-dibromobenzene-1,4-dicarbaldehyde (**DBTA**) and 2,4,6-tris(4-aminophenyl)-s-triazine (**TAPT**) under solvothermal conditions was used to obtain **Br-COF**. For the sensor assembly, a mixed of **Br-COF**, electrocatalytic La_2_O_3_, and high conducting **MWCNT**s were drop casted over GC electrode. The developed electrochemical platform was applied for the sensitive and selective detection of dopamine and uric acid with low detection limits (0.039 μM and 0.024 μM for dopamine and uric acid, respectively) and wide linear ranges (2–450 μM for dopamine and 0.4–450 μM for uric acid). Hence, it could be employed to detect simultaneously the contents of these analytes in biological and medicine samples.

Other examples of the combination of COF with a metal oxide nanomaterial is reported by Y. Chen et al. [94]. A monomer-mediated in situ growth strategy is followed for the controllable construction of the core–shell **Co_3_O_4_@TAPB-DMTP−COF** composite using **TAPB** (1,3,5-tris(4-aminophenyl)benzene) and **DMTP**, (2,5-dimethoxyterephaldehyde) as precursors. A suspension of **Co_3_O_4_@TAPB-DMTP−COF** in water is drop casted on top of a GC electrode. The modified electrode is successfully used for tert-butylhydroquinone detection at a detection limit as low as 0.02 μM (S/N = 3). Little to no interference effects from other co-existing ions allows the sensor to detect low-abundance TBHQ from complicated real samples. The role of **TAPB-DMTP-COF** is essential to improve the electrochemical stability and to ensure a longer operational time of the Co_3_O_4_ electrocatalyst.

#### 2.2.3. COFs with 2D-Nanomaterials

2D-nanomaterials have been combined with COFs in some sensor developments. In the first simpler example, a COF was combined with graphene oxide (**GO**) [95]. The **GO@COF** is obtained using benzene-1,4-diboronic acid as monomer, and it is commercially available by Baiyin COFs Chemistry Technology Co., Ltd. (Baiyin, China). GC electrode is modified by drop casting a water suspension of the COF, after which a molecularly imprinted polypirrole (MIP) is electrodeposited on the modified electrode. The developed electrochemical sensor is capable of simultaneously determining sulfadiazine and acetaminophen thanks to the selectivity granted by the MIP and the great conductivity and high porosity awarded by the **GO** and COF, respectively. Under optimal testing conditions, linear calibration curves were obtained over the concentration range of 0.5–200 μM sulfadiazine and 0.05–20 μM acetaminophen, with limits of detection being 0.16 μM and 0.032 μM, respectively.

The next example showed the combination of COF with two 2D-nanomaterials, reduced graphene oxide, and layer molybdenum disulphide (MoS_2_) nanosheets [96]. The COF (**TFPB-PDA-COF**) is synthesized by Schiff reaction between 1,3,5-tris(4-formylphenyl)-benzene (**TFPB**) and 1,4-diaminobenzene (**PDA**). The COF is mixed with amino functionalized graphene (**NH_2_-rG**) to form **COF/NH_2_-rG**. After that, the assembly of MoS_2_ and **COFs/NH_2_-rG** on GCE results in a novel **COF**/**NH_2_-rG**/**MoS_2_**/**GCE** electrochemical sensor. The use of MoS_2_ and NH_2_-rGO enhances conductivity and electrocatalysis. This methodology has been successfully used to determine the concentration of the purine bases (adenine and guanine) in thermally denatured herring sperm DNA samples with excellent results. MoS_2_ has been also used in other configuration combined with amino-functionalized carbon nanotubes@covalent organic frameworks (**NH_2_-MWCNT@COF**) [97]. **NH_2_-MWCNT@COF** nanocomposite materials enhance the electrode sensibility for sulfamerazine determination. Once **NH_2_-MWCNT@COF** is deposited by drop casting over a GC electrode, a molecularly imprinted polymer (**MIP**) membrane was anchored on the surface of this modified GC by electrochemical polymerization to achieve selective recognition for sulfamerazine. The measurements are carried out in the presence of a redox probe (ferricyanide).

#### 2.2.4. COFs with Porphyrins Derivatives

Porphyrins and metalloporphyrins have been widely used for the development of electrochemical sensors [98]. Porphyrins have many applications due to their large surface area, redox mediators, regular porosity and tuneable structures, making them suitable for detecting small molecules. The combination of porphyrins and COFs has been reported for the development of electrochemical sensors, as recently shown by Hu and co-workers [74]. The **TAPB-DMTA-COF** was used as the basic structure for the assembly of an electrochemical sensor by a one-pot method. First, they functionalized polyvinylpyrrolidone with hemin and they were able to one-pot encapsulate these particles during the COF crystallization at room temperature. Secondly, hierarchically **AuNPs** loading was accomplished by chloroauric reduction. Finally, the electrode modification was achieved by drop casting from water containing the previously dispersed **hemin**/**TAPB-DMTP-COF**/**AuNPs**. The prepared electrode is used for the sensitive and selective bisphenol A detection. The satisfactory signal amplification is based on the abundant Fe^3+^ sites of Fe-porphyrin, the high conductivity of **AuNPs** and the large specific surface area of the **TAPB-DMTP-COF**. The other example is reported by the research group of Wu Yang [99]. The synthetized COF (**CuP-SQ**) is obtained by the condensation of squaric acid (**SA**) and Copper (II) 5,10,15,20-tetrakis(4-aminophenyl) porphyrin (**TAP-CuP**) under solvothermal conditions. Drop casting of **COOH-MWCNT** colloids over a GC electrode and controlled electrodeposition of **CuP-SQ** colloids allows the building of the modified electrode named as **MWCNTs-COOH**/**CuP-SQ COF**/**GC**. Finally, electrodeposition of **CoNPs** is used to afford the modified **MWCNTs-COOH**/**CuP-SQ COF**/**CoNPs**/**GC** electrode. The resulting modified electrode is used for the simultaneous detection of guanine and adenine by DPV. Wide concentration ranges from 0.04 to 130 μM for guanine and 0.06 to 130 μM for adenine for simultaneous quantitative analysis were found. The lower detection limits were 5.5 nM and 7.2 nM (S/N = 3), respectively.

#### 2.2.5. COFs with Macrocycles

COFs can be used together with macrocyclic cationic pillar[6]arene (**CP6**) for the development of host–guest recognition sensors. In the example reported by Tan et al. [100], 2,4,6-triformylphloroglucinol (**Tp**) and 4,4′-azobisbenzenamin (**Azo**) were used as the precursors for the synthesis of a COF by following a hydrothermal synthesis process. After the synthesis of the COF, Co(II) cations are incorporated into the structure of the COF to yield **COF-Co** through a hydrothermal reaction. **CP6** can be easily adsorbed onto the surface of the COF by π−π interactions and hydrogen bond interactions to produce CP6-**COF-Co**. The composite material is drop casted on top of a GC electrode. The developed sensor is used for the determination of ascorbic acid in solutions. The Co(II) ion exhibits excellent electrocatalytic activity when **CP6** is used as the recognition element. The porous structure of the COF allows the diffusion of the species involved in the sensing process, at the time that increases the surface–volume ratio of the electrode surface, enhancing the sensor sensibility.

**Figure 3:**
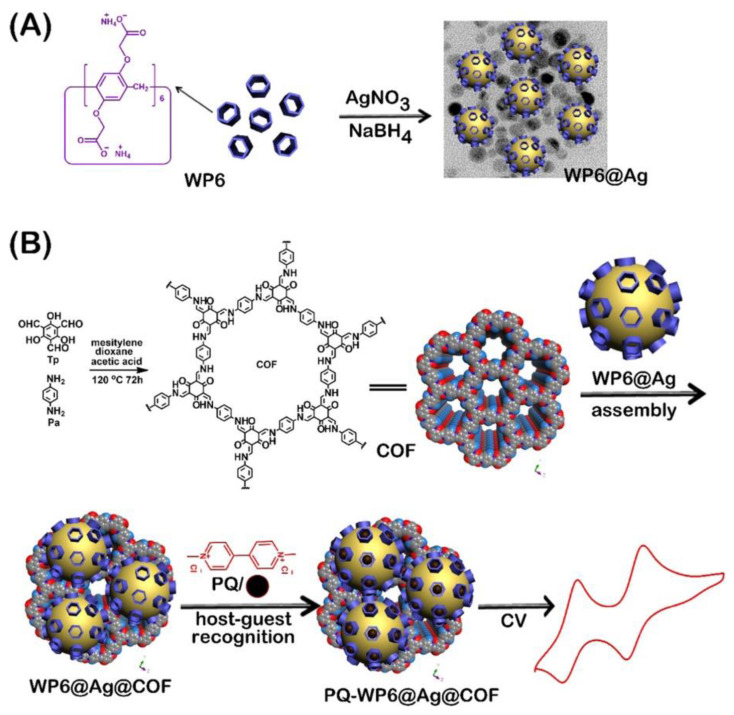
Schematic representation for the production of WP6@Ag (**A**); synthetic route to produce the COF and assembly of WP6-modified Ag nanoparticles on the surface of COF and its application for electrochemical sensing of PQ (**B**). Reprinted with permission from Ref. [58]. Copyright 2019 American Chemical Society.

Some examples have demonstrated the use of COFs as supports for metal nanoparticles, which contain the specific element for the host–guest recognition (Figure 3). This is the case of the research reported by Tan et al. [58]. In this example Ag nanoparticles (**AgNPs**) are modified with pillar[6]arene, which act as recognition element of herbicide paraquat. The modified AgNPs are loaded in the COF. In this case the synthesis of the COFs was achieved by reaction between 1,3,5-triformylphloroglucinol (**TP**) and 2,4,6-tris(4-aminophenyl)-s-triazine (**TAPT**) to yield a hexagonal β-ketoamine linked network. The researchers managed to load the **AgNPs** through a two-step sequence to enhance the COF’s conductivity in the assembly of the electrochemical sensor. First, they introduced acid functionalities in the COF by post-carboxylation to grant hydrophilicity to the pore walls. Secondly, the loading of COF with silver nanoparticles was accomplished by the in situ reduction of AgNO_3_. It should be mentioned that an abundant number of Lewis’ bases (such as N and O) introduced in the pore walls during the COF crystallization facilitated the anchoring of **AgNPs** via coordination interactions. In this way, they achieved the synthesis of a conductive composite enriched in hydrogen bonding donor/acceptor functionalities that allowed the simultaneous detection of gallic acid and uric acid. Glassy carbon (GC) electrodes are modified with the obtained composite material. Paraquat establishes host−guest interactions with pillar[6]arene adsorbed on the **AgNPs**. The Ag nanoparticles play an electrocatalytic role in sensing paraquat showing two redox processes corresponding to its reduction, being successfully quantified by differential pulse voltammetry (DPV). Other similar examples have been reported based on COFs modified with gold nanoparticles containing dihydroxylatopillar[6]arene (**2HP6@Au**) as a recognition element [101]. The COF used for this purpose is synthesized by condensation between 1,4-diaminobenzene and triformylphloroglucinol under hydrothermal reaction conditions. The COF can be functionalized with cationic pillar[6]arene (**CP6@COF**) and both composite materials are assembled by adsorption to generate a 2D heterogeneous composite (**2HP6@Au@CP6@COF**), which is subsequently drop casted on top of GC electrodes. The prepared modified electrode has been successfully used for the detection of the explosive sodium picrate (**SP**) with a detection limit of 1.7 nM. The aforementioned **2HP6@Au** is responsible for the electrocatalytic response in sensing **SP** while **CP6@COF** acts as a prominent material for gathering and recognizing sodium picrate onto the electrode surface. The picrate can settle inside the cavity of **CP6**, showing excellent host–guest interactions.

#### 2.2.6. COFs with Molecular Imprinted Polymers

Other strategies using different host–guest receptors have also been used. Instead of using macrocycles, new set-ups are using molecularly imprinted polymers (MIP) as recognition elements together with the use of COFs and metal nanoparticles. A good example for the determination of sulfathiazole was described by Sun et al. [84,102] using a molecularly imprinted polypyrrole film. **AuNPs@COF** with good electrical conductivity was introduced on the electrode surface for signal amplification and facilitation of the electron transfer processes of the redox probe employed, CuS. The sensor exhibited excellent selectivity, due to the MIP, sensitivity, reproducibility, and repeatability.

#### 2.2.7. COF Templates

COFs have also been used to enhance electron transfer efficiency in electrochemical sensors for enantiomer recognition. One recent example has been reported by L. Wang et al. [103] that prepared a COF surrounded by Fe_3_O_4_ nanoparticles (**Fe_3_O_4_@COF**). As a working electrode, a 3D-printed nanocarbon was used. The working electrode was modified by drop casting an **Fe_3_O_4_@COF** and a bovine serum albumin (**BSA**) suspension. The **BSA** is the protein responsible for chiral sensing, while **Fe_3_O_4_@COF** increases the electron transfer in the L-tryptophan electrooxidation. The prepared **Fe_3_O_4_@COF@BSA**/**3DE** presented excellent selectivity to L-Trp compared with its isomer D-Trp, which can be explained by the favourable formation of H-bonds between **BSA** and L-Trp.

Sometimes COFs have been used as templates to obtain high porous heteroatom doped carbon materials. In these examples, COF are synthesized but the obtained material is used after a calcination treatment. The advantages of these strategies are the obtaining of a high conductive carbon material with high porosity that can incorporate homogeneous heteroatom doping the graphitic carbon structure, improving the electrocatalytic activity of the material. An example reported by Yonghai Song et al. [104] starts with the solvothermal reaction between (1,3,5-triazine-2,4,6-triyl)trianiline (**TZT**) and benzo [1,2-b:3,4-b’:5,6-b’’]trithiophene-2,5,8-tricarbaldehyde (**BTT**) to yield **COF-BTT-TZT**, which presents numerous coordinative points in its structure (S and N atoms). In a second step, Pd nanorods were synthetized by in situ reduction of Pd(OAc)_2_ in a tubular atmosphere furnace under a H_2_/Ar current. The obtained material was then drop casted on top of a GC electrode in order to obtain N,S-doped C@Pd nanorods/GC. This electrochemical platform was successfully used as a paracetamol sensor, showing a low detection limit of 11 nM and wide linear range of 33 nM–120 μM, as well as good stability, reproducibility, and selectivity.

### 2.3. COFs Chelating Properties for Anodic Stripping Analysis

The capability of COFs to include multiple functional groups in their structures allows the development of COFs endowed with chelating groups. There are several examples in the literature showing the use of COFs as chelating agents to promote the pre-concentration of metal cations for differential pulse anodic stripping voltammetry. This strategy was used by Wenzhi Li and col. [105] who developed a type of COF based in the imide condensation reaction between pyromellitic dianhydride and tris-(4-aminofenil)benzene (**PMDA-TAPB-COF**) by the solvothermal method. A carbon paste electrode was subsequently prepared by using the **PMDA-TAPB-COF**. This COF showed an excellent Pb^2+^ chelating ability, which was used to assist the pre-concentration step of differential pulse anodic stripping voltammetry for the analysis of lead. Furthermore, **PMDA-TAPB-COF** selectively chelates Pb^2+^ cations, showing only a slight influence by the presence of other cations, such as Ni^2+^, Ag^+^, Cd^2+^, Fe^3+^, Co^2+^, Sr^2+^, Ca^2+^, Mn^2+^, Cr^3+^, and Zn^2+^.

T. Zhang et al. [70] reported another example of a COF carbon paste electrode for lead detection based on the synthesized **TAPB-DMTA-COF** (**TAPB**, 1,3,5-tris(4-aminophenyl)benzene; DMTP, 2,5-dimethoxyterephaldehyde). The carbon paste electrode modification was accomplished by physically mixing the COF with graphite powder and paraffin in a mortar and the paste was packed into a glass tube. The electrode was used for voltametric determination of lead with high sensitivity, low detection limit, reproducibility, good stability, and broad linear range. All these excellent properties are a consequence of the high number of active sites, and high surface area of **TAPB-DMTA-COF**. Yongmei Zhu and colleagues [106] synthetized another example of β-Ketoamine-linked COF by condensation reaction between 2,4,6-triformylphloroglucinol and melamine (**COF-TDBA-TPA**). This COF was efficiently used for the electrochemical sensing of metallic species without the need of other additives. The modification of the glassy carbon (GC) electrode with the COF was accomplished by simple liquid phase exfoliation (LPE) followed by drop casting. The **COF-TDBA-TPA**-based electrochemical sensor was used to simultaneously detect Cd^2+^, Cu^2+^, Pb^2+^, Hg^2+^, and Zn^2+^ in drinking water.

Other example of a COF-based material with chelating properties has been reported by F. Pan et al. [107]. For the synthesis of **COF-V,** 2,5-Divinylterephthalaldehyde and 1,3,5-tris(4-aminophenyl)benzene were used as precursors. This COF was mixed with 2,2′-Azobis(2-methylpropionitrile) and trithiocyanuric acid in a post synthetic modification step, generating a COF endowed with hydrosulphonyl groups (**COF-SH**). This COF was mixed with graphene followed by the addition of Nafion and water to generate a uniform suspension after sonication. The prepared suspension was drop casted on top of the GC electrode. The presence of numerous adsorption sites (18 sulphur atoms and 30 nitrogen atoms per pore) in **COF-SH**, was beneficial for the accumulation of heavy metals, while graphene enhanced the electrical conductivity. Under the optimal conditions, the sensor simultaneously detects the presence of heavy metal ions in coastal water samples at concentrations ranging from 1 to 1000 μg L^−1^. The detection limits of Cd^2+^, Pb^2+^, Cu^2+^, and Hg^2+^ were 0.3, 0.2, 0.2, and 1.1 μg L^−1^, respectively. Furthermore, the sensor exhibited good stability after multiple uses keeping 95% of the response.

COF is also combined with a carbon nanomaterial to generate a composite material with chelating properties has been reported by J. Han et al. [108]. In this work, the **COF-BTLP-1** was prepared by condensation reaction between 1,4-benzenedithiol-2,5-diamino-hydrochloride and 1,3,5-triformylbenzene in the presence of a kenaf stem-derived macroporous carbon (**3D-KSC**). Each unit of **COF-BTLP-1** has 12 adsorption sites (6 sulphur atoms and 6 nitrogen atoms) for heavy metal ions and regular holes to facilitate their transfer. The high amount of adsorption sites for heavy metal ions generated allows the identification and capture of them selectively. As result, Cd^2+^, Pb^2+^, Cu^2+^, and Hg^2+^ can be detected simultaneously. Soil and sewage samples have been successfully analysed using this modified electrode.

**Figure 4:**
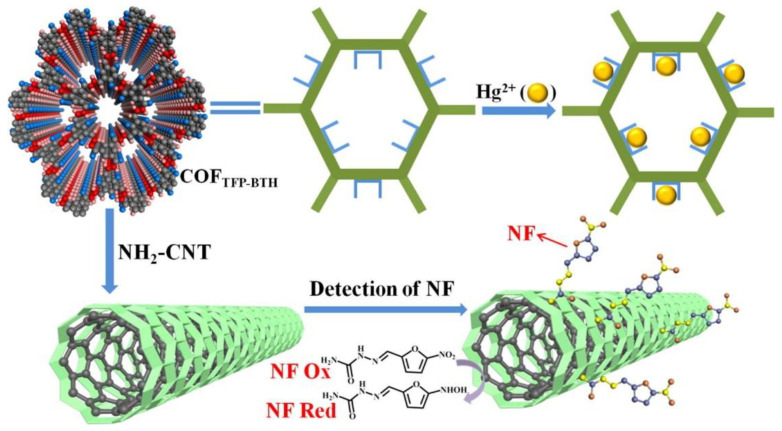
Schematic illustration of **MWCNTs@COF-TFP-BTH** showing possible adsorption sites for Hg^2+^ and nitrofural. Reprinted with permission from Ref. [109]. Copyright 2022 Elsevier.

COFs can also be used to pre-concentrate organic compounds due to the high amounts of adsorption sites present in their structures (Figure 4). A recent example has been described by Li Wang and colleagues [109] who synthetized a COF from 2,4,6-triformylphloroglucinol (**TFP**) and benzene-1,3,5-tricarbohydrazide (**BTH**) in the presence of **NH_2_-MWCNTs** to afford a **NH_2_-MWCNTs@COF-TFP-BTH**. The composite was subsequently dispersed and drop casted to afford **NH_2_-MWCNTs@COF-TFP-BTH**/GC. Nitrofural is an antimicrobial drug, which was widely used in aquaculture in the past several years. The prepared composite material **NH_2_-MWCNTs@COF-TFP-BTH** can be employed to concentrate nitrofural. Therefore, the **NH_2_-MWCNTs@COF-TFP-BTH**/GC electrode was efficiently used as a nitrofural electrochemical sensor, showing a wide linear range (9.6 nM–100 μM), good reproducibility, and stability.

## 3. Electrochemical Biosensors

COF skeletons are superior scaffolds to allow charge migration and improve the signal amplification in electrochemical sensors. The large specific surface area and tuneable pore structure of COFs makes them good candidates for the development of functional biosensors.

### 3.1. Enzymatic Biosensors

The structure tunability, porosity, crystallinity, and stability of COFs are promising characteristics for their use envisaged as host arrays for the immobilization of enzymes, among them tunability [110]. Their customizable composition through the proper choosing of their precursor, which also determines the functional groups on their surfaces, can be tailored to favour some specific interactions between them and enzymes. Furthermore, the regular distribution of nanopores in the COFs is beneficial to provide a high surface area interface for the adsorption and desegregation of enzymes and to allow a rapid movement of reagents. Moreover, the structural robustness of COFs represents an important attribute to stabilize enzymes during the biosensor manufacture process.

In the first example of a COF-based electrochemical enzymatic biosensor, a COF is able to serve as a matrix for co-immobilizing the electron mediator (1,10-dimethylferrocene, **DMFc**) and enzymes (glucose oxidase, **GOD**) onto a carbon fibre microelectrode [111]. The COF was synthesized using 1,3,5-Triformylbenzene (**TFB**) and 1,4-diaminobenzene in the presence of the carbon fibres (**CF**s) in the reaction medium as a result producing the composite material **COF-LZU1**. After that, the microelectrode is immersed in a saturated **DMFc** solution for 10 h. Finally, once the electrode is clean and dried, the microelectrode is immersed in a **GOD** solution. The resulting biosensor (**GOD**/**DMFc**/**COF-LZU1**/**CFMEs**) has been used for glucose detection in rats’ brains, being implanted on the animals’ heads. The methodology demonstrated in this study should be applicable to generalise in vivo measurements. Another enzymatic biosensor was reported by Y. Liu et al. [68]. They obtained three-dimensional kenaf stem composites (**3D-KSC**) by pyrolytic carbonization of kenaf stem (KS). Secondly, they synthetized **COF-LZU-1** by Schiff base condensation reaction between 1,3,5-Triformylbenzene and 1,4-diaminobenzene in the presence of the **3D-KSC** in order to obtain the **LZU-1-COF**/**3D-KSC** composite. Finally, acetylcholinesterase (**AChE**), graphite powder, and paraffin were mixed with the **COF-LZU-1**/**3D-KSC** to obtain the **AChE**/**COF-LZU1**/**3D-KSC** electrode (**AChE/COF-LZU1/3D-KSCE**) for identifying trichlorfon organophosphorus (OP) pesticide. The measurements were carried out in the presence of acetylthiocholine chloride, and the signal followed during the measurements is the irreversible oxidation peak of the thiocholine generated through enzymatic (**AChe**) catalysis. The presence of trichlorfon produces the inhibition of **AChe**, avoiding the generation of thiocholine, and, as a consequence, a decrease in the intensity of the oxidation wave. The enzymatic biosensor shows a wide linear range of 0.2–19 ng/mL and a low detection limit of 0.067 ng/mL.

**Figure 5:**
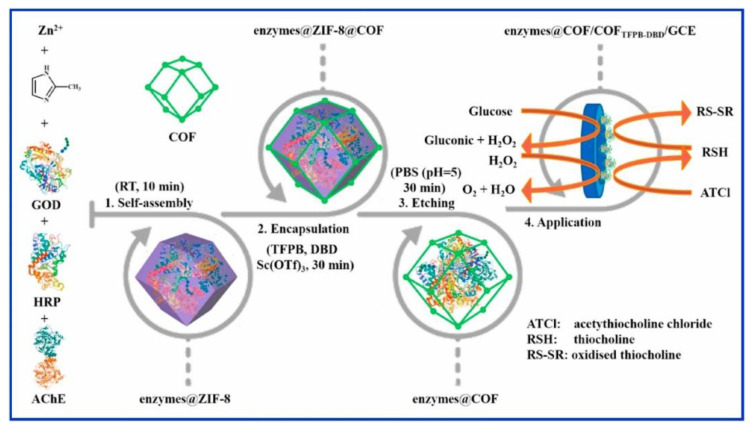
Schematic illustration of preparation and application of enzymes@COF microcapsule. Reprinted with permission from Ref. [75]. Copyright 2021 Elsevier.

Other reported works have used COFs to encapsulate multiple enzymes simultaneously (Figure 5). A representative example was reported by L. Wang et al. [112] that assembled microperoxidase-11 (**MP-11**) and **GOD** into the pores of the COF. The condensation between 4, 4, 4′′,4′′′-(ethene-1,1,2,2-tetrayl)-tetraaniline (**ETTA**) and terephthalaldehyde (**TPAL**) was used to obtain **COF-ETTA-TA** with a Kagome type lattice. The electrode modification was achieved by drop casting COF colloids on top of a GC electrode followed by the subsequent s deposition of **MP-11** and **GOD** to afford **GOD-MP-11**/**COF-ETTA-TPAL**/GC composite. Hydrogen bonds are formed between the N atoms of **COF-ETTA-TPAL** and the carboxylic groups of **GOD** and **MP-11** thus enhancing the immobilization of both enzymes. The biocompatibility of **COF-ETTA-TPAL** was good due to the lack of metal ions in the COF nanostructure. Furthermore, **COF-ETTA-TPAL** presents a 2D nanosheet structure with ordered conjugated moieties and nano-sized pores. This structure promotes fast electron transfer and enhances the mass transfer, resulting in a large specific surface area nanocomposite, with great benefits for electrochemical sensors. Regarding the biosensor detection mechanism, the O_2_ is usually used as a marker to quantify glucose given that the **GOD** modified electrode and **MP-11** can catalyse the O_2_ reduction. In this way, the intensity of the reduction peak ascribed to the O_2_ reduction decreases as the glucose concentration increases because of the high consumption of **GOD** during the transformation of glucose into gluconolactone. The developed glucose biosensor presented great performances and high selectivity for glucose determination. The reported linear range and LOD were 0.017–3 mM and 4.97 μM, respectively. Another example of multienzyme microcapsules constructed from a covalent-organic framework is reported by H. Liang et al. [75] (Figure 5). In this work, the authors synthesized a core–shell structure **enzyme@ZIF-8@COF** by the Schiff base condensation reaction. To address the synthesis of the core–shell structure, the reaction between 1,3,5-tris(*p*-formylphenyl) benzene (**TFPB**) and 4,4′-diaminobiphenyl-2,2′-dicarboxylic acid (**DBD**) was carried out in the presence of **enzyme@ZIF-8**, thus allowing the production of a composite based on a COF covalently linked to different enzymes. By using this strategy, the physical encapsulation of enzymes, such as **GOD**, horseradish peroxidase (**HRP**), and acetylcholinesterase (**AChE**), was accomplished. Finally, the modified nanoparticles were drop-casted on top of GC electrodes yield the modified electrodes. The COF’s shell with good chemical stability protects encapsulated enzymes against the external harsh environments to ultimately boost enzymatic activities. The biosensors based on the **enzymes@COF** microcapsules have been successfully used for glucose, H_2_O_2_, and malathion determination.

### 3.2. Immunosensors

COFs have also been used as a support material for the development of immunosensors. For example, a kidney injury molecule-1 (**KIM-1**) immunosensor has been recently developed [113]. A COF based on 3,5-tris(4-aminophenyl)benzene (**TAB**) and *p*-phthalaldehyde (**PTA**) was modified with AuNPs (**COFs-AuNPs**). The composite material was drop casted on top of a GC electrode and the capture antibody was immobilized on the AuNPs due to efficient amino–gold interactions. The sandwich immunosensor was completed with the addition of a secondary antibody conjugated to NiCo_2_S_4_@CeO_2_ microspheres through electrostatic interactions. The NiCo_2_S_4_@CeO_2_ microspheres provide signal amplification in differential pulse voltammetry (DPV), enhancing the signal for the H_2_O_2_ oxidation. The developed immunosensor showed high selectivity and sensitivity for **KIM-1** determination in a short analysis time.

**Figure 6:**
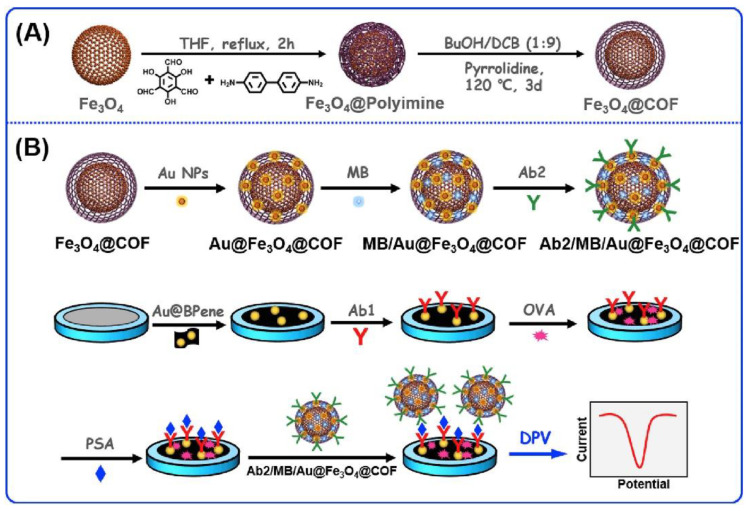
Synthesis of the **Fe_3_O_4_@COF** nanohybrid by H. Liang et al. [114] (**A**) and assembly of the immunosensor for the PSA and signal conversion strategy (**B**). Reprinted with permission from Ref. [114]. Copyright 2019 Elsevier.

COFs can be also used to develop electroactive labels linked with secondary antibodies. The high porosity together with the high amount of adsorption sites along the COFs nanostructure make them excellent candidates to guest electroactive molecules, which can be reduced or oxidized during the measuring step, obtaining an electrochemical signal proportional to the concentration of the antigen being analysed. The first example is a prostate-specific antigen (**PSA**) immunosensor developed by H. Liang et al. [114]. In a first step, as immunosensing platform, black phosphorene (**BPene**) was prepared via water-phase exfoliation. **BPene** nanocomposite (Au@BPene) was subsequently prepared by depositing Au nanoparticles (Au NPs) onto **BPene**. This nanocomposite was used as an immunosensing platform to bind primary antibodies and improve electron transfer. Regarding the labelling composite nanomaterial, they synthetized a magnetic β-ketoamine linked COF by reaction between 1,3,5-triformylphloroglucinol and 4,4′-bifenildiamine in a two-step sequence (Figure 6). First, the amorphous product was synthetized in the presence of Fe_3_O_4_ magnetic nanoparticles, which were isolated and subdued to the thermodynamic conditions to produce the reticulation processes and yield the crystalline COF. Finally, the encapsulation of Au NPS, methylene blue, and **Ab2** yielded the active bioconjugate. Once the sandwich-based immunosensor was completed, the electrochemical measurements were carried out by the DPV sensing of the electrochemical reduction of methylene blue adsorbed in the labelled nanocomposite material. The fabricated sensor exhibited linear range from 0.0001 ng/mL to 10 ng/mL, with an LOD of 30 fg/mL for the detection of **PSA**.

A similar example for **PSA** determination has been reported by J. Zheng et al. [115]. Polydopamine-coated boron-doped carbon nitride (**Au@PDA@BCN**) was used as sensing platform to attach AuNPs and immobilize primary antibodies. AuPt metallic nanoparticles and manganese dioxide (MnO_2_) were used as labelling agents to functionalize covalent organic frameworks (**AuPt@MnO_2_@COF**). The COF used was obtained by using the solvothermal method in the reaction between 1,2,4,5-tetrakis-(4-formylphenyl) benzene (**TFPB**) and 1,4-diaminobenzene (**PPDA**). Then, methylene blue was incubated with **AuPt@MnO_2_@COF**, and then the PSA affinity peptide was added, being adsorbed over the nanocomposite material. The whole material serves as a nano-catalyst and the ordered nano-pore structure allows the enrichment and amplification of the methylene blue signal molecules. DPV measurements of methylene blue reduction are carried out, showing a logarithmic relation between current intensity and **PSA** concentration. A linear range from 5 × 10^−5^ ng/mL to 10 ng/mL and a limit of the detection of 16.7 fg/mL were determined. Similar examples have been reported using COFs labelled with other electroactive molecules, such as toluidine blue (**TB**). A representative example is the development of an immunosensor for cytokeratin fragment antigen 21-1 (**CYFRA21-1**), which can be used as an important indicator for predicting non–small cell lung cancer [116]. In this work, the authors synthetized the **TpPa-NO_2_-COF** by Schiff base condensation reaction between triformylphloroglucinol and 2-nitrobenzene-1,4-diamine, loaded with AuNPs by the in situ reduction of chloroauric acid and loaded with **CYFRA21-1** antibodies (**Ab2**) and **TB**. This platform offers the possibility of dispersing numerous **Au-Ab2** assemblies and provides a large number of signal **TB** units and secondary antibodies. In this way, a sandwich-type electrochemical immunosensor was developed with AuTi_3_C_2_Tx/GC loaded with the **CYFRA21-1** antibodies (**Ab1**) for the detection of **CYFRA21-1**. The electrochemical oxidation of **TB** is followed by DPV, relating the peak current with the logarithm concentration of **CYFRA21-1**. The potential of this immunosensor for practical use has been proven by using it for determining **CYFRA 21-1** in normal human serum. To verify the accuracy and reliability of the method, five serum samples of lung cancer patients were analysed, and the results were confirmed by magnetic particle chemiluminescence assay (MPCA). On the other hand, an immunosensor for human chorionic gonadotropin (a biomarker of bowel cancer, lung cancer, or ovarian adenocarcinoma) in human serum was developed using **TB** adsorbed on COFs in the labelling agent preparation [78]. **Pa-Tp-COF** was synthesized by Schiff-base reaction between 1,3,5-triformylphloroglucinol (Tp) with *p*-phenylenediamine (Pa). Before the COF synthesis, they were able to incorporate MnO_2_ nanosheets by the room temperature reduction of KMnO_4_ in the presence of calix[6]arene (SCX6). Interestingly, the SCX6-functionalized MnO_2_ displayed great water dispersity and can be generated in the presence of the COF structure without affecting the crystallinity of the network. The COF/MnO_2_ composite was impregnated with AuNPs by simple dispersion, centrifugation, and freeze-drying yielding Au/COF/MnO_2_. Following, toluidine blue (**TB**) was incorporated via impregnation as a signal molecule for the electrochemical sensing. Finally, the specific binding bio-site was added also by mixing the **TB**/Au/**Pa-Tp-COF**/MnO_2_ with anti-HCG (**Ab2**) to obtain the bioconjugate solution of **Ab2**/**TB**/Au/**Pa-Tp-COF**/MnO_2_. In parallel to this, a GC electrode was modified with AuNPs and halloysite nanotubes (**HNTs**). Following, to block non-specific binding, ovalbumin solution was incubated to yield a **Ab1**/Au/eHNT modified electrode. Finally, HCG and **Ab2** bioconjugate solutions were incubated to yield HCG/OVA/Ab1/Au/eHNT-modified electrode. Following this green and facile method, the authors construct a sandwich-type COF-based carrier–analyte–electrode selective sensor, taking advantage of the hierarchical structure of the COF to incorporate both electroactive and bio-site moieties. Measurements were carried out in the presence of hydrogen peroxide, which oxidises **TB**. DPV measurements were used to correlate the peak current for the reduction of **TB_ox_** with the logarithm concentration of human chorionic gonadotropin.

A different strategy to obtain a labelling agent is the use of COFs together with peroxidase enzymes, which are adsorbed by the COFs nanostructures. This strategy has been followed by S. Feng et al. [117] during the development of cardiac troponin I (cTnI) immunosensor. They managed to crystallize the **HRP-Ab2-Au-COF** nanocomposite material in a three step-method. First, the COF crystallization was performed by the Schiff base condensation reaction between the 1,3,5-Tris-(4-aminofenil)benzene (**TAPB**) and the 2,5-dimethoxyterephaldehyde (**DMTA**) at room temperature in acetonitrile, to afford the **TAPB-DMTA-COF** as a spherulite polymorph with an average diameter centred at 256 nm. In a second step, AuNPs loading was achieved by suspending COF in water followed by the subsequent chloroauric acid reduction with sodium borohydride similar to that described by Shirong Hu and col. [118]. It is worth pointing out that the generation of AuNPs did not affect the COF morphology as revealed by the microscopy analysis. Finally, the electrochemical immunosensor was assembled through a Au-NH_2_ reaction between AuNPs and secondary antibody (**Ab2**) and horseradish peroxidase (**HRP**). The synergistic effect between the high AuNPs conductivity and the high dispersion due to the COF’s intrinsic porosity makes this composite an ideal platform for selective target recognition and signalling. The gold-working electrode was modified by adsorbing the specific capture antibody (**Ab1**) and the subsequent block of the surface with **BSA**. DPV measurements were carried out in a solution containing hydroquinone as redox mediator and H_2_O_2_, which is employed as substrate by the HRP enzyme to oxidized hydroquinone. The electrochemical immunosensor showed a wide linear range from 5 pg/mL to 10 ng/mL for the detection of cardiac troponin levels (**cTnI**), with a detection limit of 1.7 pg/mL, demonstrating good selectivity and reproducibility.

### 3.3. Genosensors

**Figure 7:**
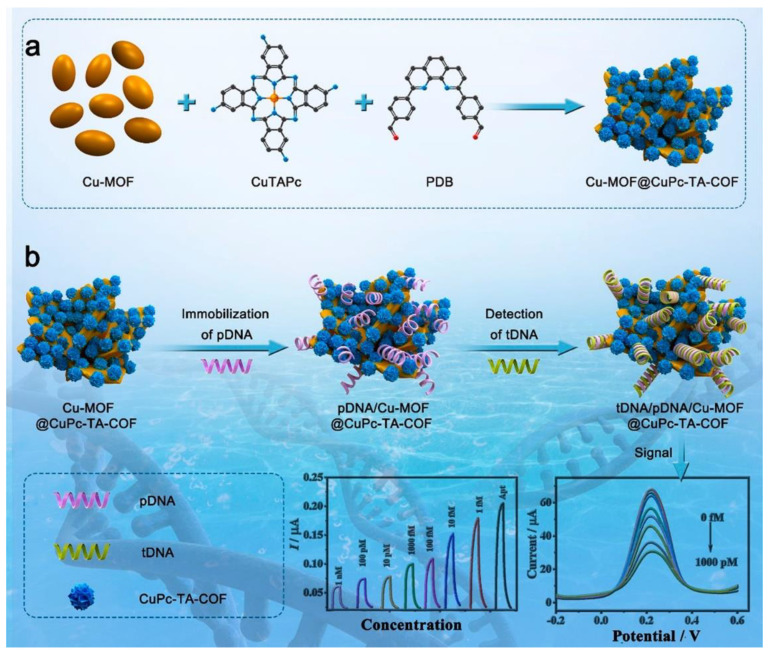
(**a**) Preparation of the **Cu-MOF@CuPc-TA-COF** Hybrid Material by the Miao Du’s research group [119] and (**b**) Construction Procedure of the **HIV-1 DNA** Biosensor Based on the **Cu-MOF@CuPc-TA-COF** Hybrid. Reprinted with permission from Ref. [119]. Copyright 2021 American Chemical Society.

COFs can be used as structural materials to adsorb oligonucleotides and their further use as DNA probes for the development of genosensors. A hybrid MOF/COF nanocomposite material was developed by Miao Du’s research group [119] (Figure 7). The developed nanocomposite material has been used on a label free genosensor for **HIV-1 DNA** probe. They developed the synthesis of a phtalocyanine-based COF by the Schiff base condensation reaction of copper-phthalocyaninetetra-amine (**CoPc-TA**) and 2,9-bis[*p*-(formyl)phenyl]-1,10-phenanthroline in the presence of a Cu-MOF, yielding the **Cu-MOF@CuPc-TA-COF** dual-structure. Electrode modification was performed at variable percentages of the **Cu-MOF@CuPc-TA-COF** colloids by drop casting onto a GC electrode to obtain a **Cu-MOF@CuPc-TA-COF**/GC electrode. **Cu-MOF@CuPc-TA-COF** acts as the sensitive platform for anchoring the **HIV-1 DNA** probe strands and can be used as the signal transducers for EC biosensors. The electrochemical detection is carried out by DPV, using ferrocyanide as redox probe. The current intensity decreases when increasing amounts of **HIV-1 DNA** target sequence is retained by hybridization with the DNA probe immobilized on the **Cu-MOF@CuPc-TA-COF**. The genosensor has been successfully used for the detection of **HIV-1 DNA** in human serum, demonstrating good agreement with real concentrations. Another example of genosensor using COF in the electrochemical platform has been reported by L. Guo et al. [120]. The novel biosensor was applied for the non-small cell lung cancer of circulating tumour DNA (NSCLC ctDNA) detection with acceptable stability, reproducibility, and specificity. **COF-TAPB-TFPB** was synthesized 1,3,5-tris(*p*-formylphenyl) benzene (**TFPB**) and 1,3,5-tris(4-aminophenyl) benzene (**TAPB**). After that, nitrogen-doped graphene (**NG**) is suspended together with polyethyleneimine (**PEI**), and then is mixed with **COF-TAPB-TFPB**, generating a **NG-PEI-COF_TAPB-TFPB_** composite material. GC electrode is modified by drop casting a suspension of **NG-PEI-COF_TAPB-TFPB_**. Secondly, AuNPs are drop casted on top of the modified electrode, and the specific DNA capture probe is incubated, being immobilized due to the formation of Au–N bonds. As a label element, **Fe-MOF** with amine groups was synthesized according to [121]. AuNPs were bound to the surface of **Fe-MOF** through Au–N bonds (**Fe-MOF@AuNPs**). **Fe-MOF@AuNPs** was incubated with the probe for DNA detection. The DNA target probe is incubated over a capture Probe-NG-PEI-COF-TAPB-TFPB/GC electrode, and after that, the label element is incubated. Finally, after the typical sandwich hybridization, the electrochemical detection is carried out in the presence of K_4_[Fe(CN)_6_], which is transformed into Prussian blue (**PB**) on the surface of the electrode due to the Fe^3+^ provided by the label element. The signal obtained by DPV for the electrochemical oxidation of **PB** was finally related with the concentration of the DNA target probe.

### 3.4. Aptasensors

Similar to that shown above for genosensors, COFs can be also used as supports to adsorb oligonucleotides, which are capable of recognizing target proteins with an affinity and specificity rivalling that of antibodies [122].

Zhongyi Liuc and col. [123] obtained a porphyrin-based COF (**pCOF**) by Schiff base condensation reaction between 5,10,15,20-tetrakis(4-aminophenyl) porphyrin (TAPP) and terephthaldehyde (TA). Gold electrodes were modified by drop casting a suspension of **pCOF**. The authors used **pCOF** as platform to immobilize the label-free epidermal growth factor receptor (EGFR)-targeting aptamer stands (Apt) by non-covalent interactions. The porous structure of the **pCOF** allowed the functionalization of the aptamer beyond the particle outside surface, allowing an increase to the surface area of the accessible binding sites with living cells. Furthermore, the **pCOF** displayed high charge carrier mobility and good electrochemical activity even after functionalization. The **pCOF**-based aptasensor benefits from the C=N- and secondary-NH_2_ groups, as well as from the macromolecular structure of **pCOF**. It possesses a π-conjugated network, and its electrons are expected to migrate quickly into the pCOF layers. The developed aptasensor has been used to directly determine an epidermal growth factor receptor (EGFR) protein, as well as living Michigan cancer foundation-7 (MCF-7) cells. The measurements were carried out by electrochemical impedance spectroscopy (EIS) and DPV using [Fe(CN)_6_]^3−/4−^ as a redox probe showing low LOD of 5.64 fg/mL and 7.54 fg/mL, respectively. The determined linear range was 0.05–100 pg/mL. For the detection of living MCF-7 cells, the aptasensor showed an LOD of 61 cell/mL and a linear range of 500 × 10^5^ cell/mL. Another example of a cancer biomarker aptasensor was developed by Jiangnan Li et al. [124] from melem and hexaketocyclohexane octahydrate. The M-HO-COF was suspended and then drop casted on top of a gold electrode (AuE). Then, the M-HO-COF/AuE was incubated with the aptamer solution. The M-HO-COF network is composed of C=N and highly conjugated aromatic moieties and presents large pore size, high surface area, and excellent bio-affinity toward aptamer strands. Vascular endothelial growth factor 165 (VEGF_165_)-targeted aptamer is anchored to M-HO-COF through intermolecular forces. EIS was used during the measurements using ferricyanide as redox probe. A bi-functional aptasensor for the detection VEGF_165_ and K7M2 cells has been developed. The aptasensor showed appropriate selectivity toward other biomarkers or normal cells, good stability, acceptable reproducibility, and applicability.

**Figure 8:**
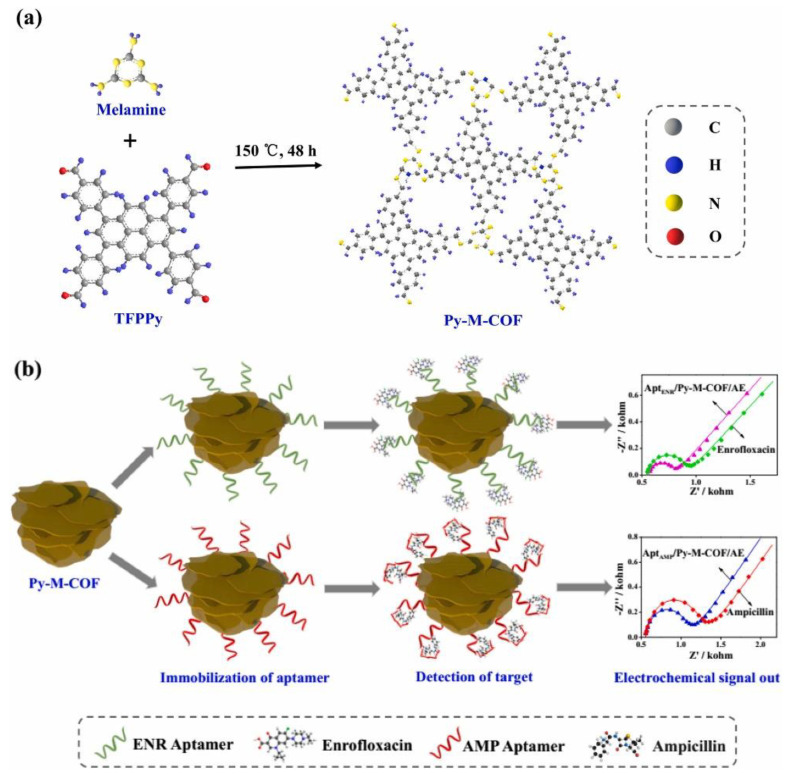
(**a**) Scheme of the synthesis of **Py-M-COF**. (**b**) Scheme of electrochemical detection of ENR and AMP using the **Py-M-COF**-based aptasensors. Reprinted with permission from Ref. [125]. Copyright 2019 Elsevier.

Antibiotics have also been the target of aptasensors based on COFs, as shown in a recent study reported by M. Wang et al. [125]. To address the synthesis of the COF network, 1,3,6,8-tetrakis(4-formylphenyl)pyrene was reacted with melamine in DMSO as a solvent to afford the Py-M-COF as a powder (Figure 8). Liquid phase exfoliation of Py-M-COF in water allowed the modification of Au-electrodes via drop casting. Finally, the modified electrodes were immersed in aptamer-saturated solutions of enrofloxacin (ENR) and ampicillin (AMP) separately and dried under a N_2_ flow affording the Apt/Py-M-COF/AuE. Because of the high amounts of the functional groups, the high specific surface areas, and the pore cavities of the COF porous frameworks, important amounts of aptamer strands can be immobilized on the Py-M-COF. EIS measurements showed that Py-M-COF-based aptasensors presented good sensing performances and low LOD of 6.07 and 0.04 fg/mL for detecting ENR and AMP, respectively.

The combination of COFs with carbon nanomaterials has been also used for the development of aptasensors as recently reported by He and co-workers [126]. For this purpose, a COF was synthesized using 1,3,5-tris(4-aminophenyl)benzene and 2,5-dimethoxyterephthalaldehyde as precursors in the presence of amino carbon nanotubes. The composite material suspension was drop casted on top of a Au electrode. The modified AuE was incubated in an aptamer phosphate buffered saline (PBS) solution. The sensing performance was mainly attributed to the mass of aptamers immobilized on the COF through π–π stacking and hydrogen bond interactions and the great electrical conductivity of the CNTs. Atrazine, an extensively used artificial herbicide, has been detected using EIS, showing an LOD of 0.67 pg/mL. Real water samples from rivers and taps were also successfully analysed. Another example of the combination of COFs with carbon nanomaterials was described by M. Sarabaegi et al. [127]. In this example, the COF is obtained from the reaction between 2,4,6-triamino-1,3,5-triazine and trimesic acid. Polyacrylonitrile (**PAN**) was mixed with the COF using DMF as a solvent. After that, an electrospinning device was used to generate a composite nanomaterial made of nitrogen-doped carbon nanofibers and the COF (**COFCNF**). **COFCNF** was dispersed in DMF and drop casted on top of the GC electrode. The **COFCNF** was functionalized with mercaptopropionic acid (**MPA**), and then used to impregnate the fibres. **EDC**/**NHS** was employed to activate the carboxylic groups. The aptamer probe was attached to the **COFCNF** through the formation of covalent bonds given that amide bonds stimulate amine aptamer groups to **COFCNF**/GCE carboxyl groups. The proposed aptasensor was used for tyrosinamide determination. Quantifying tyrosinamide in the blood serum as a risk factor for diabetes can be very helpful in treating and preventing this issue. The measurements were carried out using EIS. The aptasensor present an LOD of 0.53 pM, linearity from 0.0016–0.08 nM, and 0.08–9 nM.

COFs have also been used in combination with metal nanoparticles for the development of aptasensors. Metal nanoparticles can supply the limited electrical conductivity of COFs. T. Zhang et al. [128] reported an aptasensor for the target cancer biomarker thrombin (**TB**) detection. The cyclotrimerization of benzene-1,4-diboronicacid (**BDBA**) yielded a hexagonal boroxine-linked COF. Subsequent liquid phase exfoliation and drop casting of the COF-colloids over a GCE yielded a COF/GCE modified electrode, which was loaded with AuNPs by in situ reduction of chloroauric acid and anchored to the capture probe (**TBA 1**). Finally, BSA incubation yielded the **BSA**/**TBA 1**/Au-COFs/GCE modified electrode. The authors used a sandwich-type sensor by the incorporation of Thrombin binding aptamers (**TBA 2**) to the **Au@ZIF-8-**(**NiPd**) composite. The amount of **TB** was determined by measuring the peroxidase activity of **TBA 2–Au@ZIF-8-**(**NiPd**) using **TMB**, **ABTS**, and **OPD** as the chromogenics in the presence of H_2_O_2_. The synthetized **BSA**/**TBA 1**/**Au-COFs**/GCE and **TBA 2–Au@ZIF-8-**(**NiPd**) bioconjugates were used for **TB** determination in real serum samples.

COFs have been also combined with MOFs for the development of aptasensors. A recent study reported by Zhihong Zhang and col [59] started with the synthesis of a triazine based COF through nitrile cyclotrimerization reaction of 1,4-dicyanobenzene in melt ZnCl_2_ al 300 °C. The subsequent in situ formation of **Co-MOF** yielded a **Co-MOF@TPN-COF** nano-architecture. Drop casting a dispersion of **Co-MOF@TPN-COF** on top of a Au electrode yielded the modified gold electrode (AuE). Finally, the immersion of AuE into the aptamer solution afforded the final electrode with the DNA-strand anchored to the nanostructure walls. The multi-layered **Co-MOF@TPN-COF** nanosheets exhibit a high specific surface area, nitrogen-rich groups, and excellent electrochemical activity. As a result, large amounts of aptamer strands are retained on the **Co-MOF@TPN-COF** nanosheets due to the strong π–π stacking and hydrogen bonds. The developed aptasensor uses EIS to obtain the signal response, which is specific to ampicillin (**AMP**). The LOD of 0.217 fg/mL of **AMP** and a linear range between 1.0 fg/mL to 2.0 ng/mL were obtained. Siyu Lu’s research group developed an alternative strategy that involved the incorporation of Ce-MOF by in situ formed MCA-COF nanosheets at different weight ratios [76].

These nano-layers were synthetized according to a bottom-up method, which involves the reaction between melamine and cyanuric acid. Finally, for the fabrication of the **Ce-MOF@COF** based aptasensor, a dispersion of the composite in deionized water was drop casted on top of an electrode, dried, and immersed in aptamer solutions. Oxytetracycline has been successfully determined by EIS, showing an LOD of 17.4 fg/mL and a linear range between 0.1 and 0.5 ng/mL. Another example showed the use of (4-aminophenyl)benzene (**TAPB**) and 2,5-imethoxyterephaldehyde (**DMTP**) to generate a COF-based material coating a microporous MOF (**UiO-66-NH_2_**) [129]. The obtained material (**UiO-66-NH_2_@COF**) was dispersed into ethanol and drop casted on top of a gold electrode. This strategy to prepare MOF@COF core–shell composites is based on the Schiff base reaction between aldehyde group of monomers and the amine groups present on the MOF. The **UiO-66-NH_2_@COF** core–shell composite can be rationally designed and prepared using microporous **UiO-66-NH_2_** as core and mesoporous **TAPB-DMTP-COF** as shell. The modified electrode **UiO-66-NH_2_@COF**/AuE was incubated with the aptamer oligonucleotide sequence. The proposed strategy was used for two different aptamer sensors, using different specific oligonucleotides sequences for adenosine triphosphate (**ATP**), important physiological energy source for human body, and chloramphenicol (**CAP**), widely used in the therapeutic field and that can cause several health problems in human beings. EIS was used to obtain the signal response after the modified electrodes were incubated with the corresponding analyte.

In the last example, a COF-based material was used in the preparation of a label agent. The aforementioned 2,6-diaminoanthraquinone (**DA**) and 1,3,5-triformylphloroglucinol (**Tp**) were used together with Fe_3_O_4_ nanoparticles to generate [**Fe_3_O_4_@COF**(**TpDA**)] to provide **WP5A@Au@COF@Fe_3_O_4_**. After that, the oligonucleotide used as aptamer was incubated with the nanocomposite material. Finally, methylene blue (**MB**) was accommodated into **WP5A**, giving a result of **MB@Apt@WP5A@Au@COF@Fe_3_O**_4_. The biosensor construction was carried out using GC electrodes. AuNps were electrodeposited on the electrodes (Au@GCE), then the electrode was incubated with peptides, and finally blocked using 1-hexanethiol (**HT**). The immobilized peptide is specific for human norovirus (**HuNoV**). The amount of **HuNoV** was correlated with the DPV oxidation peak of **MB** retained on the label agent **MB@Apt@WP5A@Au@COF@Fe_3_O_4_**. The described biosensor detects unprocessed specimens without nucleoid acid extraction and amplification. Accordingly, this biosensor has the potential application in **HuNoV** detection in real samples and point of care testing (POCT). The fabricated biosensors were used for **HuNoV** detection in spiked oyster, strawberry, and faecal samples to explore their practical application in real samples.

Table 1 contain examples of COFs application in different real samples including sensors and biosensors. 

## 4. Conclusions

COFs are new and emerging materials with multiple application fields. Among them, sensor and biosensor development is a wide area of the application of these materials, as a consequence of their extraordinary properties. In particular, it is worth highlighting their high porosity, appropriate pore sizes for the immobilization of specific recognition elements, and post-modification capacity. In addition, the possibility of predesigning chemical structures according to the correct selection of their precursors is opening the door to interesting sensors and biosensors developments. COF can be used in the sensor platforms in order to improve specific area, serving as a supportive material for electrocatalysts, enzymes, antibodies, oligonucleotides, etc. COF can also have a specific function related to their structure, acting as an electrocatalyst, recognizing elements, and offering the functional groups needed for specific adsorption or the chelating process. Innovative strategies are using COFs as label agent developments, linking them with specific antibodies, aptamer, enzymes, oligonucleotides, etc. The great variety of COFs structures and the enormous amount of new COF designs would have a starring role in the next generation of electrochemical sensors and biosensors. Finally, the prospects of sensor assemblies should address the problems produced from the inherent insolubility of the COFs. Thus, the use of dynamic covalent chemistry to produce CONs with monomodal distributions through bottom-up strategies may improve the sensor quality, since the top-down strategies may be very destructive, yielding the uncontrolled distribution of nanosheets sizes. Furthermore, to ensure a sustainable future, the inclusion of metallic additives on the sensor composition should be avoided. In addition, other additives could produce pore blockage limiting the analyte diffusion to the active sites. Furthermore, the characteristic electrical insulator behaviour of COFs must be addressed by either developing conductive COFs or by using few-layer COFs, ensuring close contact between COFs and electrodes. Finally, the new trends on tailor-made COFs by 3D-printing techniques and shapable monolithic aerogels will pave the way for the production of tailor-made sensors based on COFs to ensure proper device integrations and scalability.

## Figures and Tables

**Table 1:** Examples of COFs application in different samples.

COF	Modifier	Analyte	Sample	Electrode	LOD	Linear Range	Sensitivity	Ref.
DQTP-COF	β-ketoenamine	Bisphenol A, and S	Food packages	Graphite	0.15 and 0.15 µM	0.5–30 and 0.5–30 µM	0.239 and 0.150 µA/µM	[80]
TPA-COF	Composite Carbon Black	Dopamine	Medical injections	Glassy Carbon	0.17 µM	20–1000 µM	0.023 µA/µM	[65]
TAPB-DMTP-COF@PANI	polyanyline	Acetaminophen	Tablets, human blood, serum and urine	Glassy Carbon	0.032 µM	0.10–500 µM	0.1229 µA/µM	[84]
Fe_3_O_4_@AT-COF	Fe_3_O_4_	*p*-nitrophenol and o-nitrophenol	Lake and tap water	Magnetic beads	0.2278 and 0.5925 μM	10–3000 and 10–3000 µM	0.7588 and 0.7799 µA/µM	[85]
FeTAPP-TA-COF	Composite graphene aerogel	NO	Complex biological system	Glassy carbon	0.030 μM	0.18–400 μM	8.8 μA/μM·cm^2^	[86]
MA-TP-COF	β-Ketoamine	Cd^2+^, Cu^2+^, Pb^2+^, Hg^2+^ and Zn^2+^	Drinking water	Glassy Carbon	0.922, 0.450, 0.309, 0.208 and 0.526 nM	-	17.8, 36.6, 53.2, 79.1 and 31.3 μA/μM cm^2^	[106]
DTPA-TFB-COF	kenaf stem-derived macroporous carbon	Cd^2+^, Pb^2+^, Cu^2+^ and Hg^2+^	Soil and sewage		12.3, 11.8, 18.6 and 21.4 nM	0.0369–18.0, 0.0356–19.0, 0.0536–19.0, and 0.0503–18.0 µM	1337.4, 1389.0, 886.2 and 770.0 μA/μM cm^2^	[108]
GOD/DMFc/PA-TFB-COF/CFMEs	DMFc and GOD	Glucose	Rats’ brains	Carbon fibre microelectrode	0.36 µM	1.08 μM to 8.5 mM	46.55 mV/mM cm^2^	[111]
enzyme@ZIF-8@COF	GOD, HRP, AChE	Glucose, H_2_O_2_ and malathion		Glassy Carbon	0.85 μM, 2.81 nM, 3.0 × 10^−13^ g/L	2.83 μM–8.0 mM, 9.53 nM–7.0 μM, 10^−12^ g/L–10^−8^ g/L	-	[75]
COFs-AuNPs	AuNPs, Capture antiKIM-1	KIM-1	Plasma samples	Glassy Carbon	2.00 fg/mL	0.01–50.00 pg/mL	1.8981 µA·mL/pg	[113]
AuPt@MnO_2_@COF	AuNPs, PSA affinity peptide	PSA	Human serum		16.7 fg/mL	0.00005–10 ng/mL	2.237 µA/log (ng/mL)	[115]
TP-PANO_2_-COF	AuNPs, CYFRA21-1 antibodies	CYFRA21-1	Human serum		0.1 pg/mL	0.5–1.0 × 10^4^ pg/mL	6.3 µA/log (pg/mL)	[116]
Cu-MOF@Cu-PcTA-COF	Cu-MOF, HIV-1 DNA probe strands	HIV-1 DNA	Human serum	Glassy carbon	0.07 fM	1 fM to 1 nM	-	[119]
**Acronyms**								
1,3,5-triformylphloroglucinol (**TP**) and 2,6-diaminoanthraquinone (**DQ**)								
**TPA-COF** triphenylamine-based covalent-organic framework								
**TAPB**, 1,3,5-tris(4-aminophenyl)benzene; **DMTA**, 2,5-dimethoxyterephaldehyde								
1,3,5-tris(4-aminophenyl) benzene (**TAPB**) and 1,3,5-benzenetricarboxaldehyde (**TFB**)								
5,10,15,20-tetrakis [(4-aminophenyl) porphinato]-iron (**Fe-TAPP**) and terephthalaldehyde (**TA**)								
2,4,6-triformylphloroglucinol and melamine (**MA-TP-COF**)								
1,4-benzenedithiol-2,5-diamino-hydrochloride (**DTPA**) and 1,3,5-triformylbenzene (**TFB**)								
1,3,5-Triformylbenzene (**TFB**) and 1,4-diaminobenzene (**PA**)								
1,3,5-tris(*p*-formylphenyl) benzene (**TFPB**) and 4,4′-diaminobiphenyl-2,2′-dicarboxylic acid (**DBD**) in the presence of **enzyme@ZIF-8**								
Triformylphloroglucinol and 2-nitrobenzene-1,4-diamine (**PANO_2_**)								
Copper-phthalocyaninetetra-amine (**Cu-PcTA**) and 2,9-bis[*p*-(formyl)phenyl]-1,10-phenanthroline (**PDB**)								

## Data Availability

Not applicable.
